# Coordinate effects of P2X7 and extracellular acidification in microglial cells

**DOI:** 10.18632/oncotarget.24331

**Published:** 2018-01-29

**Authors:** Ponarulselvam Sekar, Duen-Yi Huang, Shwu-Fen Chang, Wan-Wan Lin

**Affiliations:** ^1^ Graduate Institute of Medical Sciences, Taipei Medical University, Taipei, Taiwan; ^2^ Department of Pharmacology, College of Medicine, National Taiwan University, Taipei, Taiwan

**Keywords:** P2X7, ATP, acidification, mitochondrial respiration, mitochondrial fission

## Abstract

Extracellular adenosine 5′-triphosphate (ATP) is a damage-associated molecular pattern and contributes to inflammation associated diseases including cancer. Extracellular acidosis is a novel danger signal in the inflammatory sites, where it can modulate inflammation, immunity and tumor growth. Extracellular acidification was shown to inhibit P2X7-mediated channel currents, while it remains unknown how acidification and P2X7 together affect cellular responses. Here, we treated BV-2 microglial cells with ATP in a short period (<15 min) or a sustained acidified condition. For short acidification we compared the actions of neutralized ATP and acidic ATP in a condition with pH buffering. For sustained acidification, we treated cells with neutralized ATP in acidic medium or acidic ATP in medium without pH buffering. In the short acidified condition, neutralized ATP induced higher responses than acidic ATP to increase intracellular calcium and reactive oxygen species, decrease intracellular potassium and induce cell death. In contrast, these cellular responses and mitochondrial fission caused by neutralized ATP were enhanced by pH 6.0 and pH 4.5 media. P2X7 activation can also rapidly block mitochondrial ATP turnover and respiration capacity, both of which were mimicked by nigericin and enhanced by acidity. Taken together P2X7-mediated ionic fluxes and reactive oxygen species production are attenuated under short acidification, while sustained acidification itself can induce mitochondrial toxicity which deteriorates the mitochondrial function under P2X7 activation.

## INTRODUCTION

Microglia are the resident immune cells in the central nervous system (CNS) [1]. In response to brain injury or immunological stimuli, these cells become activated and migrated to the site of injury and secrete numerous chemokines, reactive oxygen species (ROS), and pro-inflammatory cytokines. Microglia also actively involve in the phagocytosis of apoptotic cells and microbes. Although microglial activation is essential to eliminate pathogenic agents and prevent neuronal damage, it is a double edge sword during chronic inflammatory conditions such as neurodegenerative disease and CNS infections [2, 3].

P2X7 is a ligand-gated ion channel receptor and is ubiquitously expressed in all types of cells. Among P2 purinoreceptor subtypes, P2X7 is usually activated by high concentration of ATP (> 500 μM) in a free acidic ATP4^−^ form [4, 5]. Thus extracellular ATP that can be confined locally at sites of damaged tissues is regarded as an endogenous damage associated molecular pattern (DAMP). Activation of P2X7 rapidly triggers Na^+^, K^+^ and Ca^2+^ ions across the plasma membrane, which is followed by increasing membrane permeability for larger organic cations and forming a large, non-selective pore allowing molecules with molecular weight up to 900 Da to enter the cells [5, 6]. The large pore formation consequently initiates several cellular events, including activation of the NLRP3 inflammasome, opening of pannexin 1 and connexin hemichannels, membrane blebbing, ROS production, loss of mitochondrial membrane potential, and eventually cellular death [7–9]. P2X7 is highly expressed in both macrophages and microglia [10], and is critical for protective innate immune responses during the early phases of microbial infection [11]. Nevertheless, pro-inflammatory and pro-apoptotic actions of P2X7 contribute to the chronic inflammation and pathogenesis of various diseases, including arthritis, inflammatory bowel disease, neurodegenerative diseases, chronic pain, mood disorders and cancers [12–18].

Acidic environment frequently exists at the site of inflammation, representing a novel endogenous danger signal to alert the innate immunity [14, 15, 19]. In tissues with chronic inflammation, the pH values within the carotid atherosclerotic plaques, synovial fluid in rheumatic joints, and exhaled breath condensate of patients with acute asthmatic attack may be decreased to 6.8~5.2 [19–21]. Lower pH in the inflammatory loci mainly results from the activation of recruited immune cells, which undergo aerobic glycolysis and secrete more lactic acid to meet the higher energy demand [22, 23]. Studies have demonstrated that acidic extracellular environment can modulate apoptosis, phagocytosis and trans-differentiation of neutrophils [24, 25], regulate gene expressions [26, 27], control phenotypic change and voltage-gated K^+^ channels in astrocytes [28], impair P2Y receptor-induced migration in microglia [29], amplify proatherogenesis [30] and nociceptive responses [31]. Notably, NLRP3 inflammasome can sense acidic condition and trigger pH-dependent caspase-1 activation and IL-1β secretion. In contrast, extracellular alkaline condition strongly inhibits the IL-1β response to several known NLRP3 activators [32].

It is well known that inflammation can be induced by pro-inflammatory mediators and DAMPs [13]. Given that ATP and acidic pH are independent DAMPs, which can be concomitantly induced at the inflammatory loci, their combined action is an interesting issue to be investigated. To date only one study showed that the maximal ionic current response of P2X7 is suppressed by extracellular acidification, and five amino acid residues in the extracellular domain of P2X7 contribute to the inhibitory effect of acidification [33]. In this study, we applied an *in vitro* cell model to investigate the interplay of P2X7 agonists (ATP and BzATP) and acidic pH in BV-2 microglial cells. To this end, we designed two acidification modes, i.e. short time and long lasting acidification. For short time acidification, we treated cells with neutralized ATP (nATP) or acidic ATP (aATP). The latter gives an acidified solution and return to pH 7.4 when cells were kept in CO_2_ incubator within 15 min. For long lasting acidification, we kept cells in NaHCO_3_-free complete media with pH adjustment and maintained in an incubator without CO_2_ input. We found that extracellular acidification exerts dual actions to coordinate ATP-induced intracellular calcium increase, ROS production, mitochondrial dysfunction and cell death. Short acidified condition is sufficient to inhibit P2X7-mediated cellular responses, while long lasting acidified condition exerts an additive effect with P2X7.

## RESULTS

### Neutral ATP induced higher cellular responses than acidic ATP

**Figure 1:**
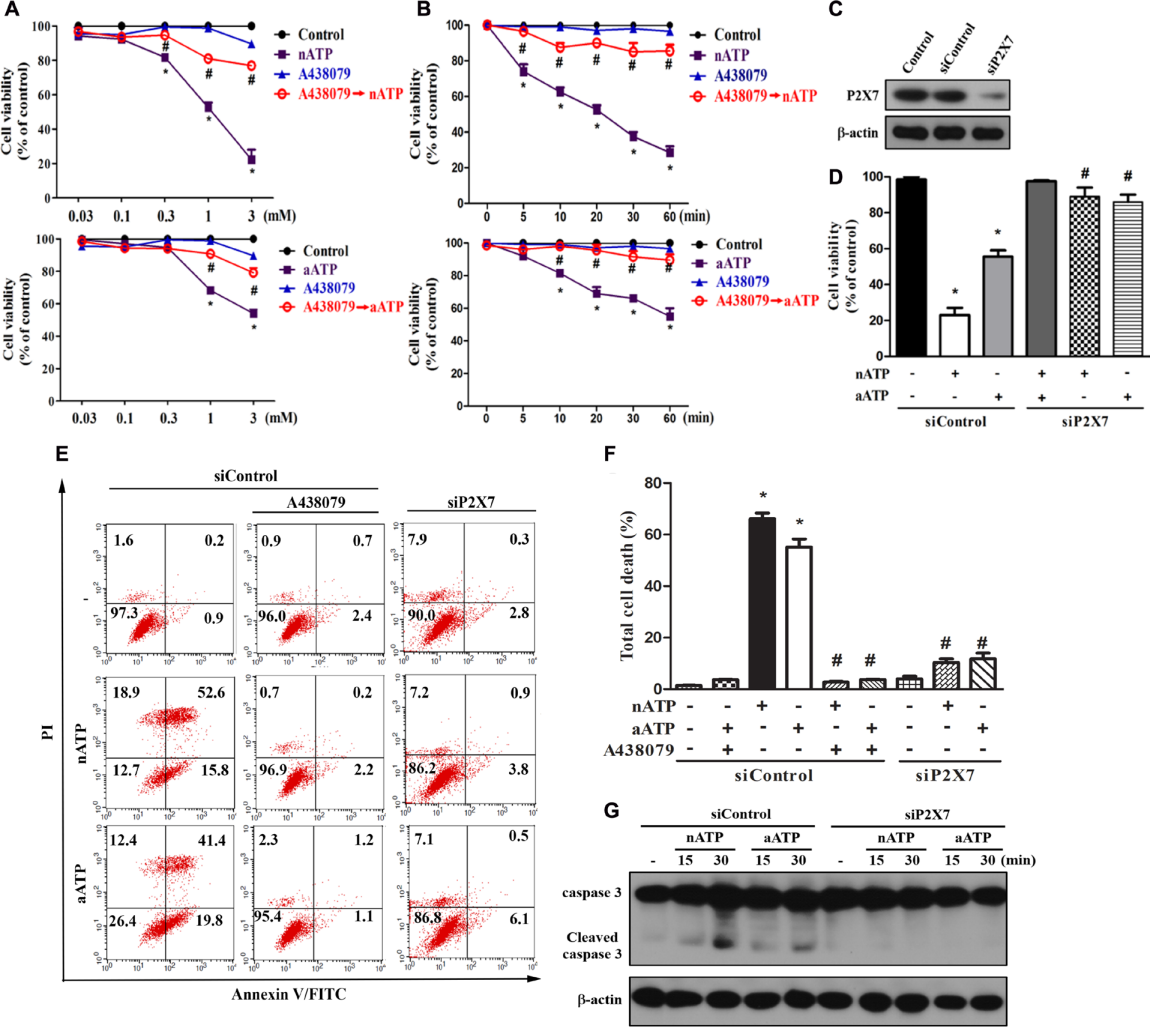
Neutral ATP caused more prominent cell death than acidic ATP via P2X7 (**A**, **B**) BV-2 microglial cells were pretreated with A438079 (10 μM) for 30 min prior to the stimulation of aATP or nATP at different concentrations for 1 h (A) or at 3 mM for different time points (B) as indicated. (**C**–**G**) BV-2 cells were transfected with siControl or siP2X7 for 48 h. After transfection, cells were treated with A438079 (10 μM) or ATP (3 mM) for 1 h (D–F) or indicated time (G). Cell viability was determined by MTT assay (A, B, D) or annexin V/PI staining (E, F). Total lysates were subjected to SDS-PAGE followed by immunoblotting (C, G). For all immunoblotting data, β-actin was detected as an internal control. Data were the mean ± S.E.M. from at least three independent experiments. ^*^p < 0.05, indicating the significant effects of nATP and aATP; ^#^*p* < 0.05, indicating the significant effects of A438079 and silencing P2X7 to antagonize the death effect of ATP.

Due to the rapid recovery (within 15 min) of acidic pH of aATP-containing medium to neutral condition in CO_2_-buffering incubator, we regarded the different responses of nATP and aATP as the effect of short time acidification on P2X7 response. In cell viability MTT assay, we found that both nATP and aATP can induce the concentration-dependent cell death, with nATP of higher potency and efficacy. Within 2 h of incubation, the IC_50_ value of nATP was 0.92 ± 0.10 mM, and aATP at the highest concentration tested (3 mM) only induced 45 ± 3% cell death (Figure 1A). The cell death induced by nATP and aATP (each at 3 mM) was 80% and 50%, respectively (Figure 1A). Moreover, nATP treatment led to cell death with earlier onset than aATP (Figure 1B). We also found that pre-treatment of P2X7 selective antagonist A438079 (Figure 1A, 1B) and silencing P2X7 by siRNA approach (Figure 1C, 1D) can block the cell death induced by nATP and aATP, suggesting the involvement of P2X7 in extracellular ATP-induced cell death.

To further address the death mode, we conducted annexin V/PI staining. Results revealed that both forms of ATP at 3 mM dramatically and rapidly increased cell population with annexin V^+^/PI^+^ staining, suggesting a mixed cell death mode (apoptosis and necrosis) is induced. Similar to the results of MTT assay, nATP at 3 mM induced higher degree of cell death than aATP (Figure 1E, 1F). Likewise, A438079 and siP2X7 can abrogate the death caused by nATP and aATP. Accordingly, nATP induced higher extent of caspase 3 activation than aATP, and this event was blocked by siP2X7 (Figure 1G).

**Figure 2:**
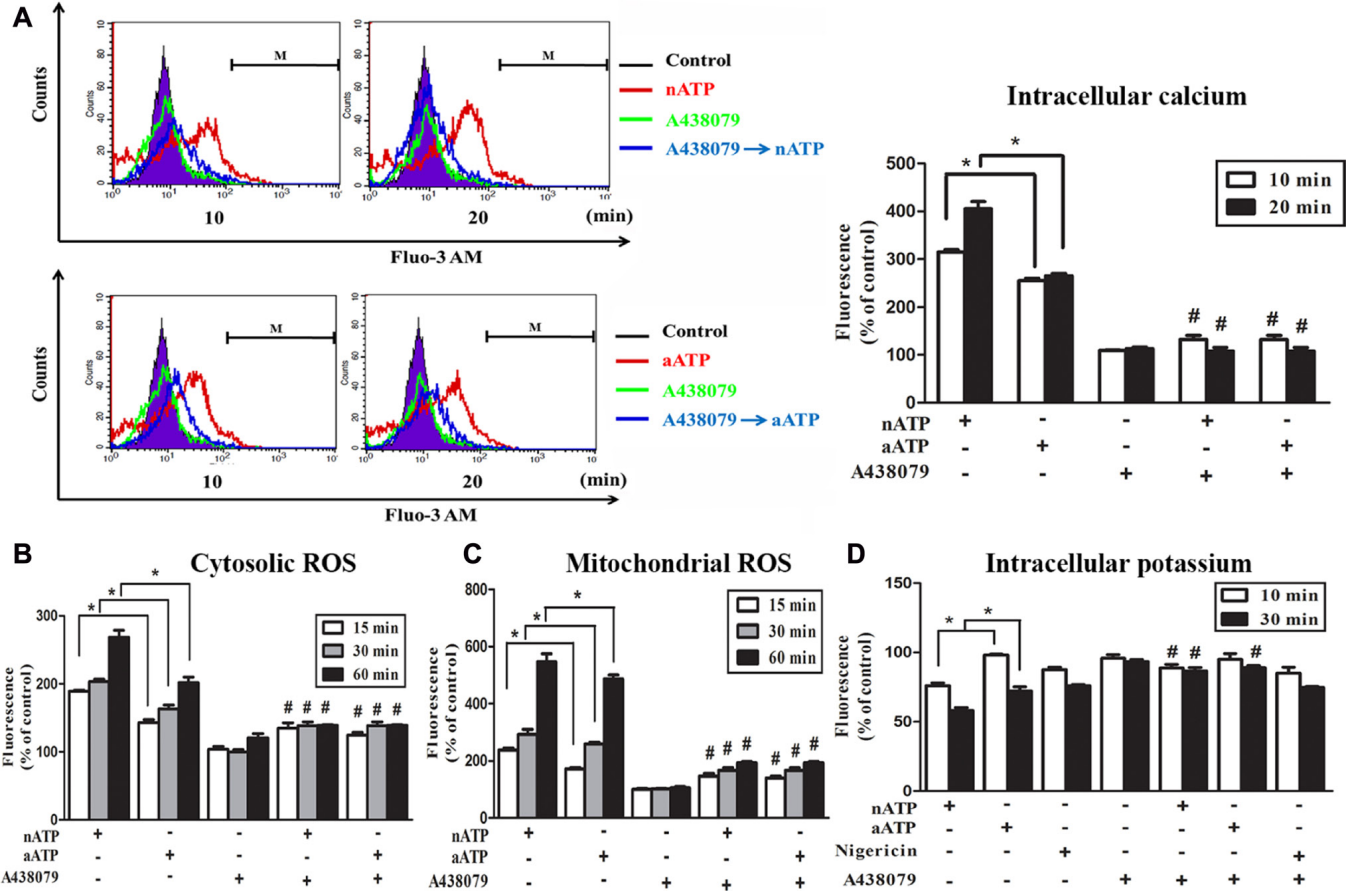
Effects of nATP and aATP on intracellular calcium, potassium and ROS BV-2 cells were pretreated with A438079 (10 μM) for 30 min and then stimulated with nATP (3 mM), aATP (3 mM) or nigericin (10 μM) for indicated time points. After treatment, cells were labelled with 1 μM Fluo-3 AM (**A**), 5 μM DCFDA (**B**), 5 μM MitoSOX (**C**) or 5 μM apg-2 (**D**) for the measurements of intracellular calcium, cytosolic ROS, mitochondrial ROS and intracellular potassium, respectively. The fluorescence intensity of the control group was set as 100% to express the relative values. Results were the mean ± S.E.M. from 3 independent experiments. ^*^p < 0.05, indicating the higher effects of nATP than aATP to increase intracellular calcium and ROS, but decrease intracellular potassium; ^#^*p* < 0.05, indicating the significant antagonistic effect of A438079 on ATP responses.

Next we used Fluo-3 AM to monitor the [Ca^2+^]i. We found that a rapid increase of [Ca^2+^]i was evoked by nATP and aATP (each at 3 mM) within 20 min, and the response of nATP was significantly higher than that of aATP. Likewise, A438079 co-treatment abolished the increase of [Ca^2+^]i (Figure 2A). The weaker effects of aATP than nATP on cell viability and [Ca^2+^]i increase suggest that short acidification can attenuate the ATP-induced P2X7 activation. We further compared the effects of nATP and aATP on intracellular ROS production and intracellular potassium. As shown in Figure 2B and 2C, both forms of ATP can increase cytosolic and mitochondrial ROS production in a time-dependent manner, and nATP triggered higher induction level within 15–60 min than aATP. Similarly, nATP induced a P2X7-dependent potassium efflux with faster onset and greater extents than aATP (Figure 2D). The selectivity of A438079 on P2X7 was further tested with nigericin, which is a potassium ionophore known to partially mimic the cellular actions of exogenous ATP through P2X7. Our data revealed that nigericin-elicited potassium efflux was unaltered by A438079 (Figure 2D).

### Sustained acidification enhances nATP-induced calcium increase and ROS production

**Figure 3:**
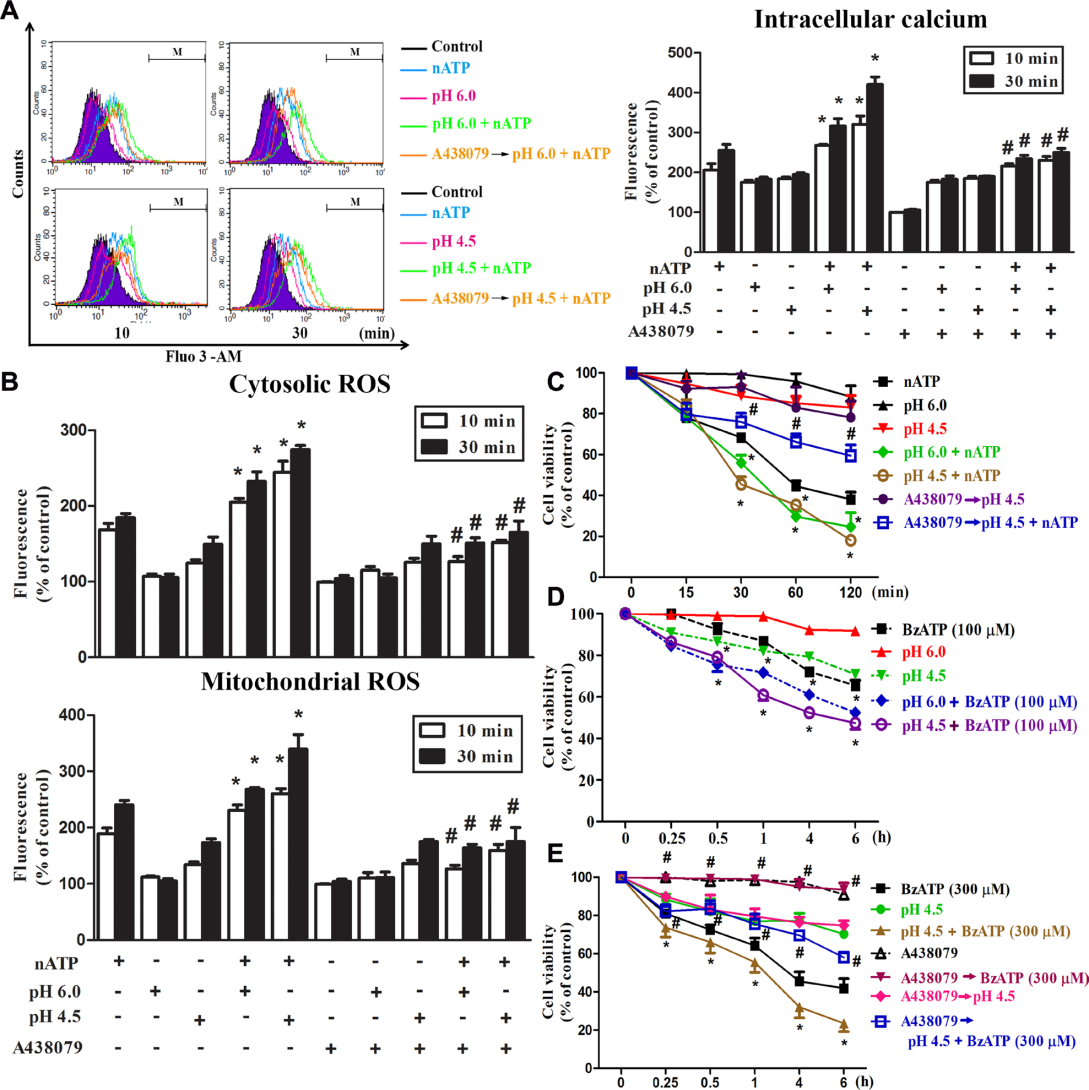
Coordinated effects of P2X7 and acidification on intracellular calcium and ROS BV-2 cells cultured in a complete DMEM were changed to the pH buffer-free medium (absence of NaHCO_3_) at pH 7.4, 6.0 or 4.5 and stimulated simultaneously with nATP (3 mM) or BzATP (100 or 300 μM). In some experiments, A438079 (10 μM) was pretreated for 30 min before changing the media to acidified condition and stimulation with agents. After stimulation for indicated time points, intracellular calcium (**A**), cytosolic and mitochondrial ROS (**B**) were determined by labelling cells with Fluo-3/AM, DCFDA and MitoSOX, respectively. The fluorescence intensity of the control group was set as 100% to express the relative values. In (**C**–**E**), cell viability was determined by MTT assay. Data were the mean ± S.E.M. from 3 independent experiments. ^*^p < 0.05, indicating the enhanced effects of nATP (or BzATP) and acidification in combination; ^#^*p* < 0.05, indicating the antagonist effect of A438079 on the individual action of nATP and BzATP as well as in their combination with acidification.

After we found the inhibition of P2X7-mediated responses by short time extracellular acidification, we were interested to understand the effect of sustained extracellular acidification on P2X7 activation, because sustained lower pH is commonly detected in the inflammatory microenvironment. Therefore, we treated cells in lower pH unbuffered DMEM (pH 6.0 or pH 4.5) together with nATP in 37°C incubator without CO_2_ input to prevent the change of pH. In this condition, the pH of acidic media and aATP-containing DMEM was maintained for up to 2 h, the longest time period tested (data not shown). We found that acidification itself at either pH 6.0 or pH 4.5 rapidly increased [Ca^2+^]i with similar extents at 10 and 30 min, while their responses were additive and synergistic to nATP (3 mM), respectively (Figure 3A). Furthermore, we found that sustained extracellular acidification not only increased the cytosolic and mitochondrial ROS with earlier onset of pH 4.5 than pH 6.0, but also enhanced the responses of nATP (Figure 3B). ATP-induced, but not low pH-induced changes in intracellular calcium (Figure 2A, Figure 3A) and ROS (Figure 2B, 2C, Figure 3B) were blocked by P2X7 antagonist A438079. The additive or synergistic effect of nATP and low pH was also reduced by A438079 to the level of acidification itself (Figure 3A, 3B).

With respect to cell viability, our data revealed that nATP-induced cell death was enhanced by acidification, both of pH 6.0 and pH 4.5 (Figure 3C). In addition, the synergistic effect of pH 4.5 and ATP was inhibited by A438079, which failed to reduce the effect of low pH (Figure 3C). To further verify the coordinated actions of P2X7 and acidification, we used the more specific P2X7 agonist BzATP and determined its effect on cell viability in different pH environment. We found that BzATP-induced cell death at 100 μM was enhanced by acidification, at either pH 6.0 or pH 4.5 (Figure 3D). Moreover, higher cytotoxicity was induced by 300 μM BzATP and this action was also enhanced by pH 4.5 but blocked by A438079 (Figure 3E). All these results suggest that in contrast to short period of acidification, sustained acidification can potentiate the cellular responses mediated by P2X7, implying an amplified outcome of both danger signals.

### Coordinated effects of ATP and sustained acidification on mitochondrial membrane potential and mitochondrial morphology

**Figure 4:**
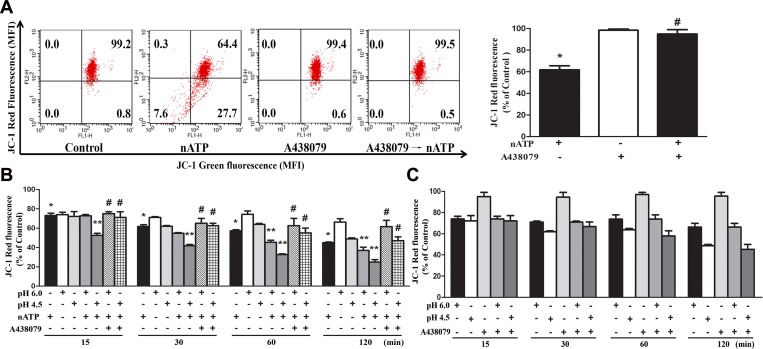
Effects of nATP, aATP and acidification on mitochondrial membrane potential (**A**) BV-2 cells were pretreated with A438079 (10 μM) for 30 min and then stimulated with nATP (1 mM) for 10 min. (**B** and **C**) Cells were incubated in NaHCO_3_-free medium of indicated pH and stimulated with nATP (1 mM) and/or A438079 (10 μM) as indicated for different time intervals. After treatment, cells were subjected to JC-1 staining for 30 min and FACS to determine the aggregation and monomeric forms of JC-1, which were represented by red and green fluorescence, respectively. Quantification of cell population with JC-1 red fluorescence was performed, and was presented as % of control in cells without treatments of drugs or low pH. Data were the mean ± S.E.M. from 3 independent experiments. ^*^p < 0.05, indicating the significant effects of nATP; ^**^p < 0.05, indicating the enhanced effect of nATP and low pH in combination; ^#^*p* < 0.05, indicating the antagonistic effect of A438079 on nATP response, either in the neutral (A) or acidified media (B).

As mitochondria are important in controlling cell viability that is instigated during P2X7 activation [34], we investigated the combinational effects of P2X7 agonist and sustained acidification on mitochondrial membrane potential (ΔΨm) and mitochondrial dynamics. Using JC-1 fluorescence probe we found that nATP (1 mM) can dramatically decrease ΔΨm and this effect was abrogated by A438079 (Figure 4A). In acidification condition, the ΔΨm loss caused by nATP (1 mM) was enhanced (Figure 4B). Meanwhile, the acidification-induced ΔΨm loss was unaltered by A438079 (Figure 4C), ruling out the involvement of endogenous ATP-P2X7 pathway in the action of acidification.

**Figure 5:**
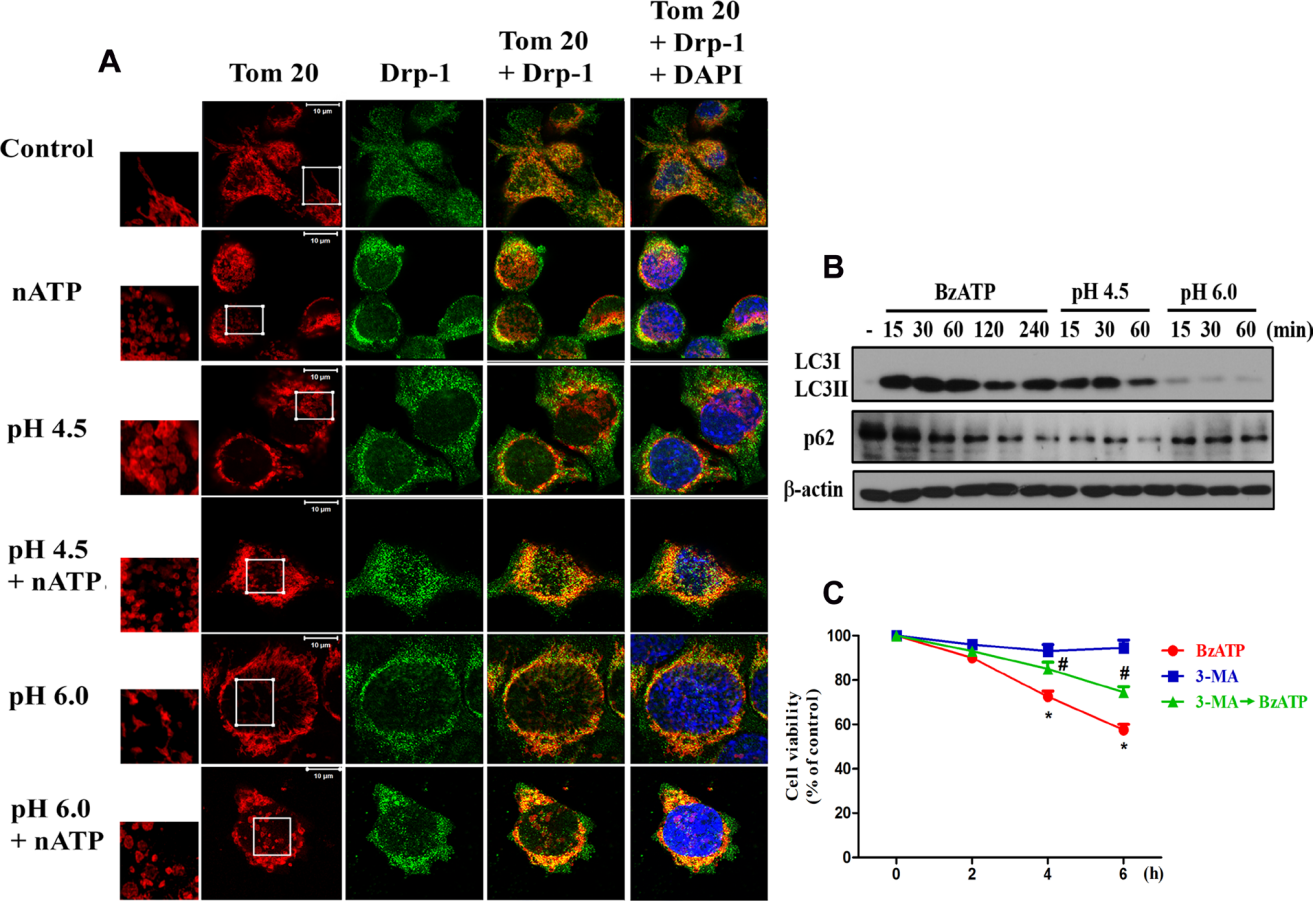
P2X7 activation and extracellular acidification changed mitochondrial dynamics (**A**) BV-2 cells were treated with the acidified media (pH 6.0 and pH 4.5) and/or nATP (1 mM) for 30 min. Cells were fixed with 4% paraformaldehyde and stained with Drp-1 antibody and Tom-20. The co-localization of Drp-1 and Tom-20 immunofluorescence indicated the mitochondrial fission. Note the changes of mitochondrial morphology from elongated shape to fragmented form after nATP and/or acidification treatments as shown by higher microscopic magnification at the indicated area. The increased co-localization of Tom-20 and Drp-1 under stress is correlated with the phenotype of mitochondrial fission. Confocal images of mitochondria were representative of three independent experiments. (**B**) BV-2 cells were stimulated with BzATP (100 μM) or incubated in low pH at indicated time periods, and then cell lysates were subjected to immunoblotting. (**C**) BV-2 cells were treated with 3-MA (5 mM) and/or BzATP (100 μM) for indicated time periods. Cell viability was determined by MTT assay. Data were the mean ± S.E.M. from 3 independent experiments. ^*^p < 0.05, indicating the significant effects of BzATP; ^#^*p* < 0.05, indicating the inhibitory effect of 3-MA on BzATP-induced cell death.

In addition to ΔΨm measurement, image data of mitochondrial morphology revealed that nATP can dramatically change the microglial shape from the resting ramified form to the active spherical form (Figure 5A). When incubating in acidic medium for 30 min, the cells swelled rapidly; and swelling phenotype at pH 6.0 condition was more obvious than pH 4.5 environment and was diminished by co-treatment with nATP (Figure 5A). With Tom-20 staining our data demonstrated the abilities of nATP and acidification to induce mitochondrial fission as indexed by the changes of mitochondrial morphology from the elongation to fragmented appearance. When using Drp-1 staining as another index of mitochondrial fission, higher amounts of Drp-1 staining co-localized with Tom-20 signal were observed after nATP and acidic pH treatment alone. An even stronger signal was obtained upon nATP treatment in acidic medium (Figure 5A). These results suggest a significant mitochondrial fission is induced by both danger signals.

After observing the mitochondrial fission induced by nATP and low pH, we further demonstrated the abilities of both stimuli to induce autophagy, as indexed by the accumulation of LC3II and downregulation of p62 (Figure 5B). In addition, the less effects of pH 6.0 compared to pH 4.5 on these events were shown. As autophagy (or mitophagy) has been reported to exert either beneficial or suppressive role in cell viability [35], we used autophagosome inhibitor 3-methyladenine (3-MA) to determine the cell death role of autophagy under P2X7 activation. Our data indicated that BzATP-induced cell death can be reduced by 3-MA (Figure 5C), suggesting the cell death effect of autophagy.

### Both nATP and acidification reduce mitochondrial respiration

**Figure 6:**
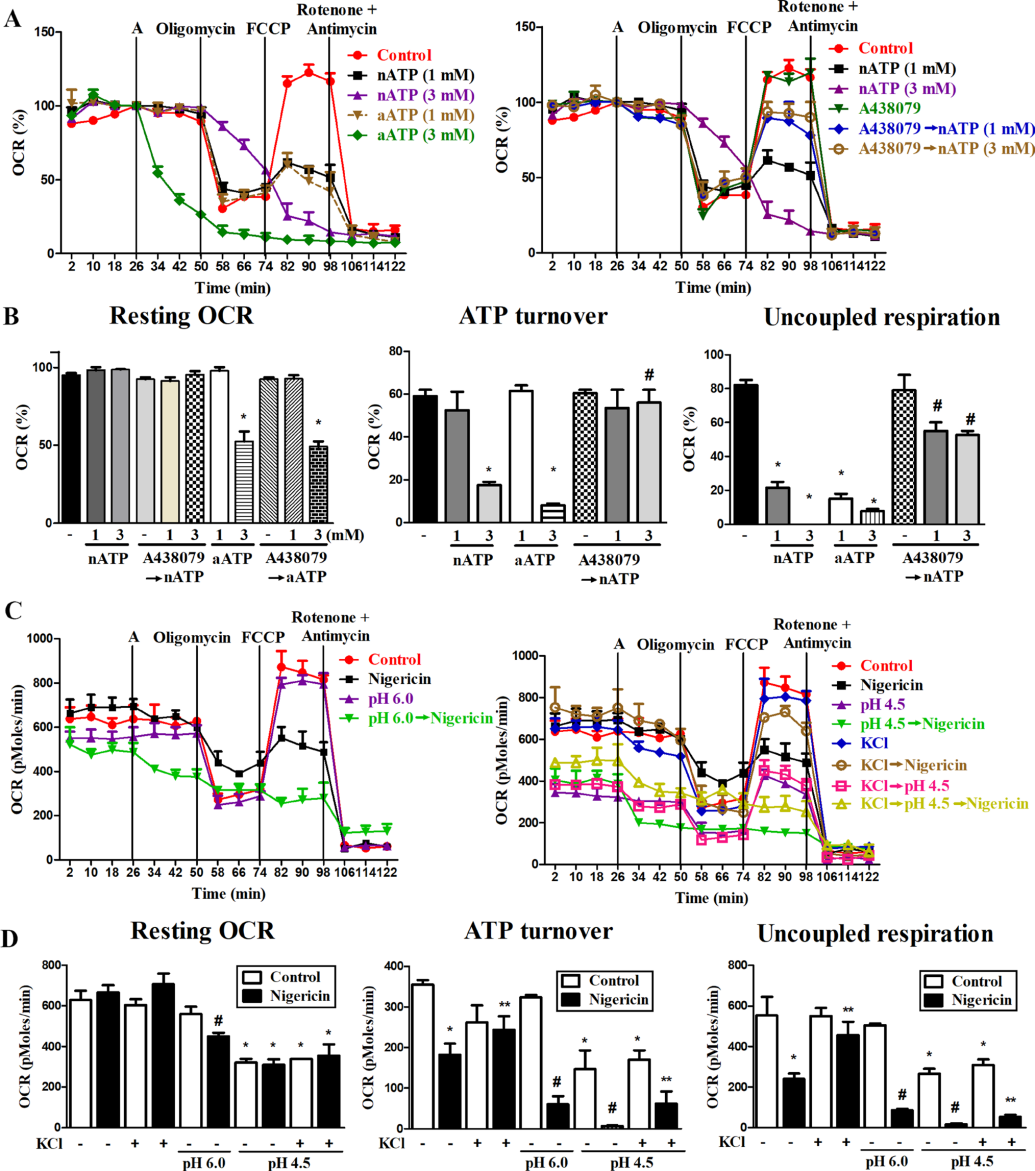
Effects of P2X7, nigericin and extracellular acidification on mitochondrial respiration (**A**, **C**) The representative effects of nATP, nigericin and extracellular acidification on oxygen consumption in mitochondria were shown. BV-2 cells were pretreated with vehicle, A438079 (10 μM), acidic media or high KCl-containing media immediately before inserting the cells in seahorse XF24 analyzer for 26 min. Then cells were treated with nATP, aATP (each at 1 or 3 mM) or nigericin (10 μM) from the port A, and subsequently oligomycin (2.5 μM), FCCP (1 μM) and rotenone (2.5 μM) plus antimycin A (2.5 μM) as indicated. (A) OCR was calculated as % of the basal respiration value at the time point before giving drug treatment from port A. (C) In order to show the individual effect of acidification, original value of OCR (pMoles/min) was presented. (**B**, **D**) The resting OCR, ATP turnover and uncoupled respiration were determined from 3 independent experiments in seahorse XF24 analyzer. ^*^p < 0.05, indicating the significant effects of nATP, aATP, nigericin and acidification alone on the specific response. ^#^*p* < 0.05, indicating the antagonistic effect of A438079 on nATP response (B) and the enhanced responses of nigericin and acidification (D). ^**^ *p* < 0.05, indicating the inhibitory effect of KCl on nigericin response (D).

We also tested mitochondrial respiration function by measuring OCR. With the aid of several agents to interrupt mitochondrial respiration chain reaction including oligomycin (an inhibitor of ATP synthase), FCCP (an uncoupling agent of electron transport and oxidative phosphorylation), rotenone (an inhibitor of electron transfer from complex I to ubiquinone) and antimycin A (an inhibitor of complex III), we determined the resting OCR, ATP turnover, and uncoupled respiration of mitochondria. Our results showed that within 24 min incubation period, although nATP (1 and 3 mM) has no significant effects on resting OCR (Figure 6A and 6B), it can attenuate ATP turnover and uncoupled respiration of mitochondria (Figure 6B). All the inhibitory effects of nATP on respiration were reversed by A438079 (Figure 6A, 6B). Notably, aATP at 3 mM led to an enormous and rapid decrease of mitochondrial respiration, ATP turnover and uncoupled respiration (Figure 6A, 6B). These dramatic reductions caused by aATP (3 mM) were not blocked by A438079 nor due to cell death (data not shown). Because OCR assay is conducted in medium without pH buffering system, the finding of different effects of aATP and nATP, in particular at 3 mM of each, is suggested to be resulting from the acidity.

Next we determined whether impairment of mitochondrial respiration caused by nATP is resulting from potassium efflux. We found that nigericin (10 μM) alone did not alter resting OCR (Figure 6C, 6D), while caused the same action as nATP to decrease the mitochondrial ATP turnover and uncoupled respiration (Figure 6D). Moreover, acidification at pH 4.5 also significantly reduced the resting OCR (Figure 6C, 6D), ATP turnover and uncoupled respiration (Figure 6D). Moreover, high extracellular KCl itself led to a modest reduction of ATP turnover, and can attenuate the inhibitory effects of nigericin but not acidity on mitochondrial respiration (Figure 6C, 6D). In acidified condition, a higher inhibition of mitochondrial oxidative phosphorylation function was detected upon nigericin treatment (Figure 6C, 6D). These results suggest that increased intracellular acidity and loss of intracellular potassium may have a coordinative effect to impair mitochondrial oxidative phosphorylation.

## DISCUSSION

P2X7 activation and functions were investigated in details over the past decade and its roles in the danger signaling with respect to intracellular potassium efflux and NLRP3 inflammasome activation have considerably given an attention very recently [6, 7]. Numerous studies have mentioned this receptor's functions in regulation of various molecular events and participation in inflammation associated diseases like arthritis and Alzheimer [36, 37]. Moreover, the role of P2X7 in microglial activation that contributes to neuronal inflammation has been demonstrated [38, 39]. Extracellular acidification is also a major cause of neuronal diseases, as the decreased extracellular pH in the brain microenvironment is common at the conditions of brain trauma, epilepsy [40] and early stage of brain tumors [41]. Moreover, acid increases inflammatory pain has been demonstrated [42]. Here we are keen to investigate the coordinate actions of both danger signals, P2X7 and extracellular acidification, in microglia. We found that acidification can exert opposite effects on regulating P2X7 action depending on the acidification periods. Acidification can inhibit P2X7-mediated intracellular stress responses at the upstream P2X7 receptor-coupled signaling level including channel currents as previously reported [33], but sustained acidification itself can exert similar effects as P2X7 to induce intracellular stress responses, which display the coordinate enhancement with P2X7 action for mitochondrial toxicity.

It is well known that ATP is a cytolytic stimulus and sustained activation of P2X7 by ATP leads to cell death in macrophages and microglia [6, 9, 43–47]. Nevertheless, the cytotoxicity induced by P2X7 is cell type dependent. Compared to previous studies in EOC13 microglia [9] and RAW264.7 macrophages [44], our data indicate that P2X7-mediated cytotoxicity in BV-2 is much remarkable. In mouse primary microglia, high concentration of ATP (5 mM) also can rapidly and significantly induce cell death as we observed in BV-2 cells [46]. In N9 microglial cells, maximal release of LDH within 6 h incubation occurred at a concentration of about 0.5 mM ATP [47]. All these findings suggest that the different cytotoxicity of exogenous ATP in microglia might depend on the expression level of P2X7 and cellular context. Besides cytotoxicity, we also observed a weak inhibition (20%) of cell proliferation after BzATP (100 μM) treatment for 6 h in BV-2 cells (data not shown). This action of P2X7 is in agreement with previous study observed in murine luteal cells [48].

In this study we demonstrated the higher extents of intracellular calcium increase, cytosolic and mitochondrial ROS production and potassium loss induced by nATP than aATP at the same concentration in BV-2 microglial cells. Consistently a higher degree of cell death accompanied by an earlier onset was observed in response to nATP than aATP. The extensive ionic fluxes and ROS production have been shown to contribute to the cell death in response to P2X7 activation in microglia [9, 10]. Previous study showed that the maximal ionic current response of P2X7 occurring within several milliseconds is suppressed by the extracellular acidified condition, and five amino acid residues in the extracellular domain of P2X7 are involved in this functional inhibition by acidic pH [33]. Because low pH of aATP-containing medium is rapidly neutralized to pH 7.4 within 15 min in CO_2_ incubator, the different extent of response caused by nATP and aATP is ascribed to the effect of short acidification on P2X7. Therefore, our current data are in agreement with previous notion indicating that the signaling of P2X7 in ionic current fluxes is negatively regulated by an acidic extracellular pH.

In this study we found that sustained acidic pH itself is a stimulus to activate microglia by altering cell shape from ramified outlook to spheroid, and to induce intracellular stress response as P2X7 activation. Extracellular acidosis can cause intracellular acidification [49, 50], calcium release from intracellular calcium stores and calcium influx [29, 51]. Notably, pH 6.0 can enlarge microglial size more apparently than pH 4.5 and this effect is reversed by P2X7 activation. Currently we do not have sufficient data to answer this finding, but cytoskeleton rearrangement by retraction of lamellipodia upon acidification of extracellular milieu as reported might be the cause [50].

Mitochondrial oxidative phosphorylation plays a crucial role in maintaining cell metabolism and viability [52, 53]. As reported, P2X7 activation causes a large increase of mitochondrial calcium and fragmentation [54]. Our findings demonstrate that P2X7 activation and intracellular acidification can individually induce mitochondrial dysfunction and display the potentiation action of each other. This notion is based on the findings of nATP and acidification to decrease mitochondrial membrane potential, induce mitochondrial fission and impair mitochondrial respiratory function. These findings can explain why 3 mM aATP can suppress mitochondrial respiration with more pronounced effect than nATP.

Recently a few studies demonstrate the relationship between acidification and mitochondrial activity. Mitochondria have outer membrane and inner membrane, and H^+^ flowing across the inner membrane into intermembrane space constitutes the electrochemical polarization of the mitochondrial membrane potential and favors the flow of H^+^ back into the matrix and couple to the ATP production. Extracellular acidification creates H^+^ on the cytosol and blocks the proton flux across the inner membrane into the intermembrane space. Moreover, extracellular acidification (lower than pH 5.5) itself can change intracellular calcium homeostasis via different processes, such as induction of calcium influx through acid sensing ion channels [55], depletion of intracellular Ca^2+^ stores [51], and alternation of the Ca^2+^ exchange function of mitochondria [56].

Because ATP and acidity are effectors present in the microenvironments of inflammatory loci, their actions associated with cancer biology have been documented. As reported, extracellular acidification is a severe pathological feature of solid tumors [57, 58]. Acidity in inflammatory tumor microenvironment is due to lactate secretion caused by anaerobic glycolysis and CO_2_ production from the pentose phosphate pathway under the hypoxia [58]. Acidic extracellular pH can modulate cancer cell metastasis through an intracellular signaling cascade that is different from hypoxia to induce the expression of certain genes of pro-metastatic factors [58, 59]. Moreover, acidic tumor milieu can modulate cancer cell metabolism and susceptibility to therapeutic efficacy [59, 60]. On the other hands, P2X7 activation is associated with a complexity of modulatory actions on tumor growth, invasiveness and release of pro-inflammatory cytokines [16, 17, 61–63]. To date, the potential interplay between P2X7 and low extracellular pH that contributes to cancer progression has not been investigated and is an interesting issue for further study.

Taken together we demonstrate the coordinate actions mediated by two endogenous danger signals in microglia. Extracellular acidification exerts different actions on P2X7 depending on the acidity periods. Short period of acidification upon P2X7 activation can target P2X7 and block the ionic responses mediated by P2X7 and the subsequent actions on mitochondrial functions. On the other hand, sustained acidification itself is an effective danger stimulus to induce intracellular stress response. The action of acidification overcomes its inhibitory action on P2X7 molecule and leads to a net potentiation effect with P2X7 activation. This study for the first time provides a detailed understanding on the coordinate actions of P2X7 and extracellular acidification in microglia, and highlights the mechanistic crosstalk of both DAMPs in regulation of inflammatory responses.

## MATERIALS AND METHODS

### Reagents and antibodies

Nigericin (Cat. tlrl-nig) was from Invivogen (San Diego, CA, USA). MitoSOX Red, Fluo 3-AM and DMEM were from Invitrogen (Carlsbad, CA, USA). ATP disodium salt hydrate, A438079, DCFDA (2′, 7′-dichlorofluorescin diacetate), BzATP, oligomycin, FCCP, rotenone, antimycin A, 3-MA and other chemicals were from Sigma-Aldrich (St. Louis, MO, USA). Asante Potassium green-2 was from Abcam (Cambridge, UK). JC-1 was from Life Technologies (Carlsbad, CA, USA). Anti-Tom 20 was from Cell Signaling Technology (Danvers, MA, USA).

### Cell culture and extracellular pH adjustment

BV-2 microglial cells were kept growing by culturing in complete DMEM supplemented with 10% fetal bovine serum (FBS), 2 mM L-glutamine, 3.7 g/l NaHCO_3_, 100 units/ml penicillin and 100 μg/ml streptomycin. In acidification experiments, the final pH of culture medium was adjusted from pH 7.4 to 6.0 or 4.5, and cells were maintained in NaHCO_3_-free complete medium (i.e. in unbuffered DMEM) and placed in an incubator at 37°C without CO_2_ after treatment with tested agents.

### Preparation of nATP and aATP

To distinguish the effects of acidification on ATP action are coming from rapid inhibition of early signaling of P2X7 as previously demonstrated in the evoked cationic channel currents [33] and/or the late-occurring cellular actions by acidification itself, we designed two acidification modes, i.e. short time and long lasting acidification. For short time acidification, we need to readjust pH from acidic condition under P2X7 activation back to pH 7.4. Nevertheless, it is not easy to change the medium pH during the experimental course without causing some unexpected interference. Because ATP disodium salt hydrate dissolving in water or NaHCO_3_-containing DMEM gives a mild acidified solution (pH 6.0 and 4.5 for 1 mM and 3 mM ATP, respectively), and ATP disodium salt hydrate solution in NaHCO_3_-containing DMEM can return to pH 7.4 when it was kept in CO_2_ incubator within 15 min, this feature allowed us to compare the cellular responses of aATP and nATP by directly treating them in BV-2 cells without subsequent pH adjustment procedure. To prepare nATP we neutralized stock aATP solution to pH 7.4 by NaOH.

### RNA interference

Mouse siP2X7 (Cat no. SC-42576) and scramble nonspecific siRNA were purchased from Santa Cruz Biotechnology (Santa Cruz, CA, USA). BV-2 cells were transfected with 100 nM siRNA by DharmaFECT Transfection Reagents (Dharmacon Research, Lafayette, CO, USA) at 50% confluence following the manufacturer's instruction. After 48 h of siRNA transfection, cells were treated with indicated drugs and then harvested for analysis.

### Western blotting

After cells were treated with specific ligands in a mentioned time points, cells were washed twice with cold PBS and then lysed using RIPA lysis buffer. After cells were harvested and sonicated, total cell lysates were centrifuged at 18,000 g at 4°C for 30 min. Protein concentrations were determined with the Bio-Rad Protein Assay. Aliquots (60 μg) of soluble protein were resolved by SDS-PAGE, and samples were then transferred onto Immobilon-P membranes (Millipore). Nonspecific binding was blocked by incubating the blots with TBST containing 5% nonfat milk for 1 h at room temperature. After incubation with the appropriate primary antibody, the membranes were washed three times with TBST and then incubated with the appropriate HRP-conjugated secondary antibody for 1 h. The membranes were washed three times with TBST, and the protein bands were detected with ECL detection reagent. To assure equal loading of samples analyzed by Western blotting, we used β-actin as an internal control.

### Measurement of cytosolic and mitochondrial ROS production

The intracellular levels of hydrogen peroxide (H_2_O_2_) and mitochondrion-specific superoxide (O_2_^−^) were measured with DCFDA and MitoSOX Red, respectively. BV-2 cells were seeded in 96-well plates for overnight followed by the indicated treatments. At the endpoint, cells were treated with DCFDA (5 μM) or MitoSOX Red (5 μM) for 30 min. The cells were then washed with PBS and collected as single cell suspensions. Fluorescence signal was detected using fluorescence spectrophotometer (Varioskan^TM^ Flash Multimode Reader, MA, USA) under the excitation and emission collected at 488 nm/520 nm and 510 nm/580 nm for DCFDA and MitoSOX Red, respectively.

### MTT assay

BV-2 cells (1 × 10^4^ per well) were plated in 96-well plates and incubated overnight at 37°C followed by the time courses as indicated above. MTT (5 mg/ml) was incubated for 1 h at 37°C, the supernatants were aspirated, and the formazan granules generated by the live cells were dissolved in DMSO. The OD values at 550 and 630 nm were measured by use of a microplate reader. The net absorbance (OD550–OD630) indicating the enzymatic activity of the mitochondria and implying cell viability was represented as 100% of the individual control.

### Annexin V/PI staining

BV-2 cells were seeded (1 × 10^7^cells/well) and incubated overnight at 37°C followed by the treatment mentioned. Following the treatment cells were suspended in an annexin V binding buffer and stained with both annexin V-FITC and propidium iodide (PI) at room temperature for 15–30 min and loaded on a flow cytometer (BD FACSCalibur, Franklin Lakes, NJ, USA) available for FL1 (annexin V) and FL2 (PI) bivariate analyses. Analysis by calculating percentage of the cells in the respective quadrants was done by using CellQuest PRO software.

### Intracellular calcium and potassium measurements

After treatment with indicated agents, the cells were loaded with l μM Fluo-3 AM (for calcium measurement) or 5 μM Asante Potassium Green-2 for 30 min at 37°C under a dark condition, followed by washing twice with PBS. Then the cells were re-suspended in 500 μl PBS, and the intracellular calcium and potassium levels were analyzed by flow cytometry.

### Measurement of mitochondrial oxygen consumption rate

the oxygen consumption rate (OCR) was measured by the Extracellular flux analyzer XF24 (Seahorse Bioscience, Houston, TX, USA). BV-2 cells were plated at 4 × 10^5^ cells/well in a Seahorse 24-well V7 microplate (Seahorse Bioscience) and cultured in normal high glucose DMEM growth medium (with NaHCO_3_, glutamine, penicillin/streptomycin and 10% FBS, pH 7.4) for 24 h in a 5% CO_2_ incubator at 37°C. Then, the medium was removed and cells were incubated in XF assay medium in the absence of NaHCO_3_ and FBS for 1 h at 37°C in measuring chamber without CO_2_ input according to the routine assay procedure of manufacturer's protocol. ATP, nigericin, ionomycin and the mitochondrial complex inhibitors [oligomycin, carbonyl cyanide-p-trifluoromethoxyphenylhydrazone (FCCP), rotenone and antimycin A] were freshly prepared in XF24 assay media. When determining the effect of acidification, high KCl and A438079 on OCR, medium of pH 4.5, pH 6.0, containing KCl (130 mM) or A438079 (10 μM) was added into wells prior to inserting the plate into the Seahorse XF24 Extracellular Flux analyzer. After 26 min to measure the basal respiration, nATP, aATP or nigericin was injected from port A; oligomycin (2.5 μM) was injected into each well at 50 min from port B, by FCCP (1 μM) at 74 min from port C and rotenone (2.5 μM) plus antimycin A (2.5 μM) at 98 min from port D. Because oligomycin is an inhibitor of ATP synthase by blocking the flow of protons through the F_0_ subunit, the decreased OCR after oligomycin treatment indicates the status of ATP turnover. FCCP is an uncoupling agent which can disrupt ATP synthesis by transporting hydrogen ions across the mitochondrial membrane instead of the proton channel of ATP synthase (Complex V). This leads to a rapid consumption of energy and oxygen without the generation of ATP. Rotenone and antimycin A co-treatment can completely interfere with the electron transport chain in mitochondria. Rotenone inhibits the transfer of electrons in complex I to ubiquinone, while antimycin A blocks the electrons from complex III. OCR was recorded as pMoles per minute, and in some cases it was calculated as percentage of the OCR value before the treatment of tested agents as we previously described [64]. All the measurements and calculations were obtained by using seahorse instrument software (seahorse bioscience) according to the manufacturer's protocol. Averages of three wells were taken per data point.

### Measurement of mitochondrial membrane potential (ΔΨm)

To investigate the mitochondrial membrane potential, BV-2 cells were seeded in 6-well plates a day before the experiment. After treatment for indicated times, the microglial cells (1 × 10^6^/ml) were incubated at 37°C for 20 min with 5 μg/ml JC-1, then washed twice before subjecting to flow cytometry. The cyanin dye JC-1 facilitates discrimination of energized and deenergized mitochondria because the normally green fluorescent dye forms red fluorescent aggregates when concentrated in energized mitochondria in response to their higher membrane potential [65]. Therefore, cell number with red fluorescence was determined as an index of mitochondrial membrane potential.

### Mitochondrial imaging

BV-2 cells were fixed with 4% paraformaldehyde at 37°C for 20 min and then permeabilized with 0.2% Triton X-100 for 15 min. After blocking with 5% BSA with normal IgG (1:300) for 1 h, immunostaining was performed with primary antibody against Tom-20 (Santa Cruz Biotechnology, CA) or Drp-1 (Abcam, Cambridge, UK) in 1% BSA overnight at 4°C. Tom-20 is a subunit of the translocase of the mitochondrial outer membrane (TOM) complex, and becomes a common marker of mitochondria. Upon induction of external stress or apoptosis, Drp-1 (Dynamin related protein-1) translocates from the cytosol to mitochondria to initiate outer mitochondrial membrane fission. After washing with PBS, cells were incubated with secondary antibody in 1% BSA (in PBS) for 1 h at room temperature and then mounted with DAPI Fluoromount-G (SouthernBiotech, Birmingham, AL). Images were acquired using a 100 X Plan-Neofluar oil objective of LSM 880 with Airyscan SR microscopy (Carl Zeiss Micro Imaging GmbH, Jena, Germany).

### Statistical analysis

Data were presented as the mean ± S.E.M. Students *t* test was used to access the statistical significance of the differences between samples, and *p* < 0.05 was considered statistically significant.

## Abbreviations

aATP: acidic adenosine 5′-triphosphate
nATP: neutralized adenosine 5′-triphosphate
ROS: reactive oxygen species
[Ca^2+^]i: intracellular calcium
H_2_O_2_: hydrogen peroxide
O_2_^−^: superoxide anion
DCFDA: 2′, 7′-dichlorofluorescin diacetat
Drp-1: dynamin-related protein-1
OCR: oxygen consumption rate
ΔΨm: mitochondrial membrane potential
FBS: fetal bovine serum
DMEM: Dulbecco modified Eagle medium
DAMP: damage associated molecular pattern
FACS: fluorescence-activated cell sorting
PBS: phosphate-buffered saline
FITC: fluorescein isothiocyanate
PI: propidium iodide
FCCP: carbonyl cyanide-p-trifluoromethoxyphenylhydrazone
TBST: tris-buffered saline with tween-20
3-MA: 3-methyladenine.

## References

[1] Nimmerjahn A, Kirchhoff F, Helmchen F (2005). Resting microglial cells are highly dynamic surveillants of brain parenchyma in vivo. Science.

[2] Garden GA, Moller T (2006). Microglia biology in health and disease. J Neuroimmune Pharmacol.

[3] van Rossum D, Hanisch UK (2004). Microglia. Metab Brain Dis.

[4] Burnstock G (2006). Historical review: ATP as a neurotransmitter. Trends Pharmacol Sci.

[5] Steinberg TH, Newman AS, Swanson JA, Silverstein SC (1987). ATP4- permeabilizes the plasma membrane of mouse macrophages to fluorescent dyes. J Biol Chem.

[6] Seeland S, Kettiger H, Murphy M, Treiber A, Giller J, Kiss A, Sube R, Krahenbuhl S, Hafner M, Huwyler J (2015). ATP-induced cellular stress and mitochondrial toxicity in cells expressing purinergic P2X7 receptor. Pharmacol Res Perspect.

[7] Schroder K, Tschopp J (2010). The inflammasomes. Cell.

[8] Hwang SM, Koo NY, Choi SY, Chun GS, Kim JS, Park K (2009). P2X7 receptor-mediated membrane blebbing in salivary epithelial cells. Korean J Physiol Pharmacol.

[9] Bartlett R, Yerbury JJ, Sluyter R (2013). P2X7 receptor activation induces reactive oxygen species formation and cell death in murine EOC13 microglia. Mediators Inflamm.

[10] Ferrari D, Villalba M, Chiozzi P, Falzoni S, Ricciardi-Castagnoli P, Di Virgilio F (1996). Mouse microglial cells express a plasma membrane pore gated by extracellular ATP. J Immunol.

[11] Coutinho-Silva R, Ojcius DM (2012). Role of extracellular nucleotides in the immune response against intracellular bacteria and protozoan parasites. Microbes Infect.

[12] Skaper SD, Debetto P, Giusti P (2010). The P2X7 purinergic receptor: from physiology to neurological disorders. FASEB J.

[13] Sperlagh B, Illes P (2014). P2X7 receptor: an emerging target in central nervous system diseases. Trends Pharmacol Sci.

[14] Garlick PB, Radda GK, Seeley PJ (1979). Studies of acidosis in the ischaemic heart by phosphorus nuclear magnetic resonance. Biochem J.

[15] Nedergaard M, Kraig RP, Tanabe J, Pulsinelli WA (1991). Dynamics of interstitial and intracellular pH in evolving brain infarct. Am J Physiol.

[16] Giannuzzo A, Saccomano M, Napp J, Ellegaard M, Alves F, Novak I (2016). Targeting of the P2X7 receptor in pancreatic cancer and stellate cells. Int J Cancer.

[17] Di Virgilio F, Adinolfi E (2017). Extracellular purines, purinergic receptors and tumor growth. Oncogene.

[18] Monif M, Burnstock G, Williams DA (2010). Microglia: proliferation and activation driven by the P2X7 receptor. Int J Biochem Cell Biol.

[19] Naghavi M, John R, Naguib S, Siadaty MS, Grasu R, Kurian KC, van Winkle WB, Soller B, Litovsky S, Madjid M, Willerson JT, Casscells W (2002). pH Heterogeneity of human and rabbit atherosclerotic plaques; a new insight into detection of vulnerable plaque. Atherosclerosis.

[20] Farr M, Garvey K, Bold AM, Kendall MJ, Bacon PA (1985). Significance of the hydrogen ion concentration in synovial fluid in rheumatoid arthritis. Clin Exp Rheumatol.

[21] Hunt JF, Fang K, Malik R, Snyder A, Malhotra N, Platts-Mills TA, Gaston B (2000). Endogenous airway acidification. Implications for asthma pathophysiology. Am J Respir Crit Care Med.

[22] Roiniotis J, Dinh H, Masendycz P, Turner A, Elsegood CL, Scholz GM, Hamilton JA (2009). Hypoxia prolongs monocyte/macrophage survival and enhanced glycolysis is associated with their maturation under aerobic conditions. J Immunol.

[23] Tannahill GM, O’Neill LA (2011). The emerging role of metabolic regulation in the functioning of Toll-like receptors and the NOD-like receptor Nlrp3. FEBS Lett.

[24] Coakley RJ, Taggart C, McElvaney NG, O’Neill SJ (2002). Cytosolic pH and the inflammatory microenvironment modulate cell death in human neutrophils after phagocytosis. Blood.

[25] Pliyev BK, Sumarokov AB, Buriachkovskaia LI, Menshikov M (2011). Extracellular acidosis promotes neutrophil transdifferentiation to MHC class II-expressing cells. Cell Immunol.

[26] Grabowski J, Vazquez DE, Costantini T, Cauvi DM, Charles W, Bickler S, Talamini MA, Vega VL, Coimbra R, De Maio A (2012). Tumor necrosis factor expression is ameliorated after exposure to an acidic environment. J Surg Res.

[27] Park SY, Kim IS (2013). Identification of macrophage genes responsive to extracellular acidification. Inflamm Res.

[28] Renner NA, Sansing HA, Inglis FM, Mehra S, Kaushal D, Lackner AA, Maclean AG (2013). Transient acidification and subsequent proinflammatory cytokine stimulation of astrocytes induce distinct activation phenotypes. J Cell Physiol.

[29] Langfelder A, Okonji E, Deca D, Wei WC, Glitsch MD (2015). Extracellular acidosis impairs P2Y receptor-mediated Ca(2+) signalling and migration of microglia. Cell Calcium.

[30] Oorni K, Rajamaki K, Nguyen SD, Lahdesmaki K, Plihtari R, Lee-Rueckert M, Kovanen PT (2015). Acidification of the intimal fluid: the perfect storm for atherogenesis. J Lipid Res.

[31] Rukwied R, Chizh BA, Lorenz U, Obreja O, Margarit S, Schley M, Schmelz M (2007). Potentiation of nociceptive responses to low pH injections in humans by prostaglandin E2. J Pain.

[32] Rajamaki K, Nordstrom T, Nurmi K, Akerman KE, Kovanen PT, Oorni K, Eklund KK (2013). Extracellular acidosis is a novel danger signal alerting innate immunity via the NLRP3 inflammasome. J Biol Chem.

[33] Liu X, Ma W, Surprenant A, Jiang LH (2009). Identification of the amino acid residues in the extracellular domain of rat P2X(7) receptor involved in functional inhibition by acidic pH. Br J Pharmacol.

[34] Nishida K, Nakatani T, Ohishi A, Okuda H, Higashi Y, Matsuo T, Fujimoto S, Nagasawa K (2012). Mitochondrial dysfunction is involved in P2X7 receptor-mediated neuronal cell death. J Neurochem.

[35] Galluzzi L, Pietrocola F, Bravo-San Pedro JM, Amaravadi RK, Baehrecke EH, Cecconi F, Codogno P, Debnath J, Gewirtz DA, Karantza V, Kimmelman A, Kumar S, Levine B (2015). Autophagy in malignant transformation and cancer progression. EMBO J.

[36] Diaz-Hernandez JI, Gomez-Villafuertes R, Leon-Otegui M, Hontecillas-Prieto L, Del Puerto A, Trejo JL, Lucas JJ, Garrido JJ, Gualix J, Miras-Portugal MT, Diaz-Hernandez M (2012). *In vivo* P2X7 inhibition reduces amyloid plaques in Alzheimer's disease through GSK3beta and secretases. Neurobiol Aging.

[37] McInnes IB, Cruwys S, Bowers K, Braddock M (2014). Targeting the P2X7 receptor in rheumatoid arthritis: biological rationale for P2X7 antagonism. Clin Exp Rheumatol.

[38] Monif M, Reid CA, Powell KL, Smart ML, Williams DA (2009). The P2X7 receptor drives microglial activation and proliferation: a trophic role for P2X7R pore. J Neurosci.

[39] Bianco F, Ceruti S, Colombo A, Fumagalli M, Ferrari D, Pizzirani C, Matteoli M, Di Virgilio F, Abbracchio MP, Verderio C (2006). A role for P2X7 in microglial proliferation. J Neurochem.

[40] Xiong ZG, Pignataro G, Li M, Chang SY, Simon RP (2008). Acid-sensing ion channels (ASICs) as pharmacological targets for neurodegenerative diseases. Curr Opin Pharmacol.

[41] Damaghi M, Wojtkowiak JW, Gillies RJ (2013). pH sensing and regulation in cancer. Front Physiol.

[42] Rocha-Gonzalez HI, Herrejon-Abreu EB, Lopez-Santillan FJ, Garcia-Lopez BE, Murbartian J, Granados-Soto V (2009). Acid increases inflammatory pain in rats: effect of local peripheral ASICs inhibitors. Eur J Pharmacol.

[43] Mackenzie AB, Young MT, Adinolfi E, Surprenant A (2005). Pseudoapoptosis induced by brief activation of ATP-gated P2X7 receptors. J Biol Chem.

[44] Noguchi T, Ishii K, Fukutomi H, Naguro I, Matsuzawa A, Takeda K, Ichijo H (2008). Requirement of reactive oxygen species-dependent activation of ASK1-p38 MAPK pathway for extracellular ATP-induced apoptosis in macrophage. J Biol Chem.

[45] Eyo UB, Miner SA, Ahlers KE, Wu LJ, Dailey ME (2013). P2X7 receptor activation regulates microglial cell death during oxygen-glucose deprivation. Neuropharmacology.

[46] Brough D, Le Feuvre RA, Iwakura Y, Rothwell NJ (2002). Purinergic (P2X7) receptor activation of microglia induces cell death via an interleukin-1-independent mechanism. Mol Cell Neurosci.

[47] Ferrari D, Chiozzi P, Falzoni S, Dal Susino M, Collo G, Buell G, Di Virgilio F (1997). ATP-mediated cytotoxicity in microglial cells. Neuropharmacology.

[48] Wang J, Liu S, Nie Y, Wu B, Wu Q, Song M, Tang M, Xiao L, Xu P, Tan X, Zhang L, Li G, Liang S (2015). Activation of P2X7 receptors decreases the proliferation of murine luteal cells. Reprod Fertil Dev.

[49] Wann JG, Hsu YH, Yang CC, Lin CS, Tai DW, Chen JS, Hsiao CW, Chen CF (2007). Neutrophils in acidotic haemodialysed patients have lower intracellular pH and inflamed state. Nephrol Dial Transplant.

[50] Faff L, Nolte C (2000). Extracellular acidification decreases the basal motility of cultured mouse microglia via the rearrangement of the actin cytoskeleton. Brain Res.

[51] Malayev A, Nelson DJ (1995). Extracellular pH modulates the Ca2+ current activated by depletion of intracellular Ca2+ stores in human macrophages. J Membr Biol.

[52] O’Rourke B, Cortassa S, Aon MA (2005). Mitochondrial ion channels: gatekeepers of life and death. Physiology (Bethesda).

[53] Szabo I, Zoratti M (2014). Mitochondrial channels: ion fluxes and more. Physiol Rev.

[54] Adinolfi E, Callegari MG, Ferrari D, Bolognesi C, Minelli M, Wieckowski MR, Pinton P, Rizzuto R, Di Virgilio F (2005). Basal activation of the P2X7 ATP receptor elevates mitochondrial calcium and potential, increases cellular ATP levels, and promotes serum-independent growth. Mol Biol Cell.

[55] Li X, Wu FR, Xu RS, Hu W, Jiang DL, Ji C, Chen FH, Yuan FL (2014). Acid-sensing ion channel 1a-mediated calcium influx regulates apoptosis of endplate chondrocytes in intervertebral discs. Expert Opin Ther Targets.

[56] Kostyuk P, Potapenko E, Siryk I, Voitenko N, Kostyuk E (2003). Intracellular calcium homeostasis changes induced in rat spinal cord neurons by extracellular acidification. Neurochem Res.

[57] Helmlinger G, Yuan F, Dellian M, Jain RK (1997). Interstitial pH and pO2 gradients in solid tumors in vivo: high-resolution measurements reveal a lack of correlation. Nat Med.

[58] Kato Y, Ozawa S, Miyamoto C, Maehata Y, Suzuki A, Maeda T, Baba Y (2013). Acidic extracellular microenvironment and cancer. Cancer Cell Int.

[59] Ippolito JE, Brandenburg MW, Ge X, Crowley JR, Kirmess KM, Som A, D’Avignon DA, Arbeit JM, Achilefu S, Yarasheski KE, Milbrandt J (2016). Extracellular pH modulates neuroendocrine prostate cancer cell metabolism and susceptibility to the mitochondrial inhibitor niclosamide. PLoS One.

[60] Dhar G, Sen S, Chaudhuri G (2015). Acid gradient across plasma membrane can drive phosphate bond synthesis in cancer cells: acidic tumor milieu as a potential energy source. PLoS One.

[61] de Andrade Mello P, Bian S, Savio LE, Zhang H, Zhang J, Junger W, Wink MR, Lenz G, Buffon A, Wu Y, Robson SC (2017). Hyperthermia and associated changes in membrane fluidity potentiate P2X7 activation to promote tumor cell death. Oncotarget.

[62] Bae JY, Lee SW, Shin YH, Lee JH, Jahng JW, Park K (2017). P2X7 receptor and NLRP3 inflammasome activation in head and neck cancer. Oncotarget.

[63] Nie J, Huang GL, Deng SZ, Bao Y, Liu YW, Feng ZP, Wang CH, Chen M, Qi ST, Pan J (2017). The purine receptor P2X7R regulates the release of pro-inflammatory cytokines in human craniopharyngioma. Endocr Relat Cancer.

[64] Wang JS, Wu D, Huang DY, Lin WW (2015). TAK1 inhibition-induced RIP1-dependent apoptosis in murine macrophages relies on constitutive TNF-alpha signaling and ROS production. J Biomed Sci.

[65] Perelman A, Wachtel C, Cohen M, Haupt S, Shapiro H, Tzur A (2012). JC-1: alternative excitation wavelengths facilitate mitochondrial membrane potential cytometry. Cell Death Dis.

